# Sulforaphane (SFN): An Isothiocyanate in a Cancer Chemoprevention Paradigm

**DOI:** 10.3390/medicines2030141

**Published:** 2015-07-17

**Authors:** Mohammad Fahad Ullah

**Affiliations:** Laboratory of Phytomedicine & Therapeutics, Prince Fahd Research Chair, Department of Medical Laboratory Technology, Faculty of Applied Medical Sciences, University of Tabuk, Tabuk-71491, Saudi Arabia; E-Mail: m.ullah@ut.edu.sa; Tel.: +966-568958324.

**Keywords:** cancer, chemoprevention, sulforaphane, adjuvant therapy, nutraceuticals

## Abstract

The International Agency for Research on Cancer (IARC) in its latest *World Cancer Report (2014)* has projected the increase in the global cancer burden from 14 million (2012) to 22 million incidence annually within the next two decades. Such statistics warrant a collaborative engagement of conventional and complementary and alternative therapies to contain and manage cancer. In recent years, there has been a shift in the cancer chemoprevention paradigm with a significant focus turning towards bioactive components of human diets for their anticancer properties. Since diet is an integral part of lifestyle and given that an estimated one third of human cancers are believed to be preventable though appropriate lifestyle modification including dietary habits, the current shift in the conventional paradigm assumes significance. Several epidemiological studies have indicated that consumption of broccoli is associated with a lower risk of cancer incidence including breast, prostate, lung, stomach and colon cancer. The edible plant belonging to the family of cruciferae such as broccoli is a rich source of glucoraphanin, a precursor of isothiocyanate sulforaphane which is considered to be a potent anti-cancer agent. Plant-based dietary agents such as sulforaphane mimic chemotherapeutic drugs such as vorinostat, possessing histone deacetylase inhibition activity. Evidence from epidemiological and experimental studies have emerged, enhancing the clinical plausibility and translational value of sulforaphane in cancer chemoprevention. The present review provides the current understanding of the cancer chemopreventive pharmacology of sulforaphane towards its potential as an anticancer agent.

## 1. Introduction

**Figure 1:**
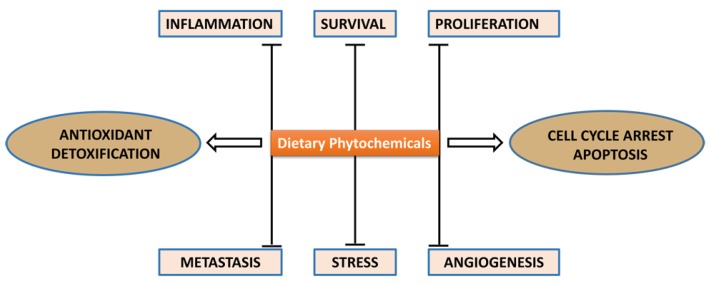
Schematic presentation of anticancer mechanisms of dietary agents in chemoprevention of cancer.

Cancer is responsible for approximately 13% of deaths worldwide (WHO 2011) and remains a growing health problem around the world particularly with the steady rise in life expectancy [1]. Lifestyle issues including poor dietary habits are major impediment in prevention of cancer. Worldwide geographical variation in cancer incidence indicates a correlation between dietary habits and cancer risk, thereby indicating dietary factors as key modulators of cancer. Recommendations based on specific dietary ingredients for both therapeutic and prophylactic interventions against various types of illness have been documented in Hippocratic and religious texts and the canons of traditional Chinese medicine [2,3]. The health benefits of a plant-based diet including potential anticancer properties are attributed to the content of bioactive phytochemicals, possessing the ability to modulate cellular antioxidant systems, enzyme-induction or inhibition, regulation of selective gene expression, interfering with cell cycle and signaling pathways, influencing the tumor microenvironment and induction of apoptosis or autophagy [4] (Figure 1). Over the years, hundreds of studies have examined the relationship between fruit/vegetable intake and cancer risk and incidence [5,6]; the majority of these have concluded that consumption of diet rich in fruits and vegetables offers a significant protective effect against cancer. Regular consumption of such diets has been shown to be protective against lung cancer; tumors of the oesophagus, oral cavity and larynx; pancreas and stomach cancers; colorectal, bladder and prostate cancers; malignancies of the cervix, ovary and endometrium; and breast cancer [7].

**Figure 2:**
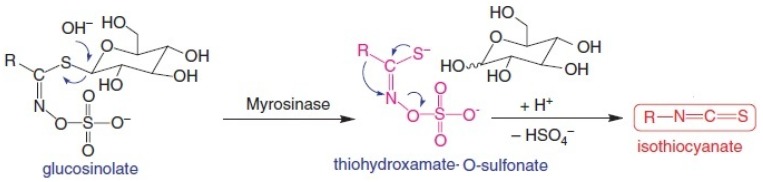
Enzyme myrosinase present in plant tissues or intestinal flora catalyses the breakdown of glucosinolates such as glucoraphanin to isothiocyanate sulforaphane.

Epidemiological studies suggest that intake of cruciferous vegetables including broccoli reduces the risks for the induction of certain forms of cancer [8,9]. This protective effect has been linked to the presence of glucoraphanin, a glucosinolate precursor of sulforaphane (SFN), an isothiocyanate (ITC) that influences the process of carcinogenesis during initiation and promotion phases [10]. It was more than 30 years ago that Wattenberg first reported inhibition of chemically induced cancer in experimental rodents upon administration of ITCs [11,12]. The study showed that the phenylethyl isothiocyanate administration, 4 h before 7,12-dimethylbenz[a]anthracene administration resulted in inhibition of mammary carcinogenesis in rats [12]. ITCs are synthesized from glucosinolates (GSs) stored in plants, upon catalytic breakdown by myrosinase (Figure 2), a thioglucoside glucohydrolase present mainly in the crucifers and released during stress or damage [13] and to some extent in the microflora of the intestinal tract [14]. The four major ITCs formed from glucosinolates by the activity of myrosinase include benzyl-ITC (BITC), allyl-ITC (AITC), phenylethyl-ITC (PEITC) and methylsulphinylbutyl-ITC (SFN). Among these, SFN was isolated from broccoli in the early 1990s as an inducer of phase 2 enzymes (xenobiotic metabolism) and since then numerous studies have proposed various anti-neoplastic pharmacological aspects of SFN, thereby suggesting its potential as a promising candidate in cancer chemoprevention [15].

Multiple action mechanisms inherent to the anticancer properties of SFN have been reported [15]. However, the three major observations specifically with regard to SFN which enhance its clinical plausibility and translational value are: (i) SFN has good bioavailability as it can reach high intracellular and plasma concentrations, and studies have reported detectable levels of SFN recorded for breast tissues after single oral administration [16,17]; (ii) In human subjects, a single ingestion of 68 g of broccoli sprouts inhibited HDAC (histone deacetylase) activity in circulating peripheral blood mononuclear cells 3–6 h after consumption, with a concomitant increase in global histone H3 and H4 acetylation—interestingly, this study provided the first translational evidence for HDAC inhibition by a natural diet “broccoli sprouts”, and support for an anti-cancer pharmacological action at intake levels readily achievable in humans [18]; and (iii) Normal cells are relatively resistant to sulforaphane-induced cell death, a characteristic of an ideal anti-cancer drug [19].

## 2. Anticancer Action Mechanisms of Sulforaphane

Components of the human diet usually have pleiotropic action mechanisms that interfere with multiple targets within the intracellular and extracellular microenvironment. Such a divergent action is advantageous against multi-factorial diseases like cancer in which multiple pathways enter into an erroneous alternative. The succeeding sections briefly outline various mechanistic approaches responsible for the cancer chemopreventive properties of sulforaphane.

### 2.1. NRF2-Mediated Elevation of Antioxidant Defense by Sulforaphane Reduces the Incidence of ROS-Induced Genomic Insult

**Figure 3:**
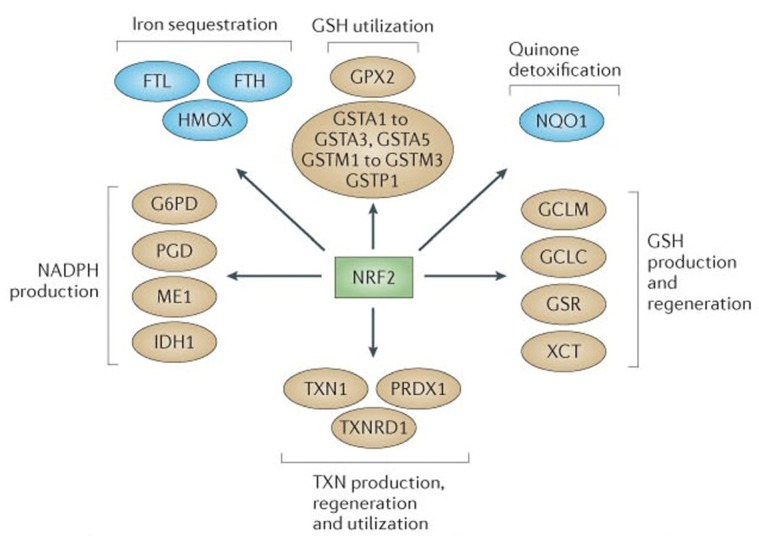
NRF2 as the master regulator of antioxidant responses*.* Nuclear factor erythroid 2-related factor 2 (NRF2) controls several different antioxidant pathways. The first is glutathione (GSH) production and regeneration, which is regulated by the following antioxidants: the glutamate-cysteine ligase modifier complex (GCLM), the GCL catalytic subunit (GCLC), the cystine/glutamate transporter XCT, and glutathione reductase (GSR). The second is glutathione utilization, which is regulated by glutathione S-transferases (GSTA1, GSTA2, GSTA3, GSTA5, GSTM1, GSTM2, GSTM3 and GSTP1) and glutathione peroxidase 2 (GPX2). The third is thioredoxin (TXN) production, regeneration and utilization which is regulated by TXN1, thioredoxin reductase 1(TXNRD1) and peroxiredoxin 1 (PRDX1). The fourth is NADPH production, which is controlled by glucose-6-phosphate dehydrogenase (G6PDH), phosphoglycerate dehydrogenase (PHGDH), malic enzyme 1 (ME1) and isocitrate dehydrogenase 1 (IDH1). Both GSH and TXN require NADPH in order to regenerate once they have reduced reactive oxygen species. These four groups of antioxidant genes,—which are all upregulated by NRF2—have both complimentary and overlapping functions. Additional antioxidants that are controlled by NRF2 include NAD(P)H:quinone oxidoreductase 1 (NQO1) and enzymes regulating iron sequestration, such as heme oxygenase (HMOX1), ferritin heavy chain (FTH) and ferritin light chain (FTL). Reproduced from the original source [20] with permission of Macmillan Publishers Ltd., United Kingdom.

One of the hallmarks of cancer cells is their dependence on increased aerobic glycolysis resulting in oxidative stress due to the accumulation of reactive oxygen species (ROS) that may directly challenge the genomic stability or participate in alterations of signaling pathways [20]. The ROS at low to moderate levels are active participants in cellular functions acting as signaling molecules that sustain cellular proliferation and differentiation, along with activating responses to oxidative stress [21]. However, excessive production of ROS damages cellular components such as DNA, proteins and lipids and serves as one of the major culprits in the induction of cancer (pre-initiation stage). ROS are constantly produced by both enzymatic and non-enzymatic reactions. Thus, a constitutive balance in the intracellular ROS status is required to maintain normal cellular homeostasis. The transcription faction NRF2 (nuclear factor erythroid 2-related factor 2) is the principal regulator of the expression of molecules with antioxidant functions within the cell [22]. NRF2 stimulates anti-stress signaling with protective response to suppress oxidative or electrophilic stress and inhibits carcinogenesis [23]. In the resting state NRF2 is inactive due to proteasomal degradation induced by a negative regulator KEAP1 (Kelch-like ECH associated protein 1). Under condition of stress, the KEAP1 is oxidized leading to the stabilization and translocation of NRF2 into the nucleus and expression of genes critical to antioxidant defense (Figure 3) [24].

Sulforaphane induces the phase II carcinogen detoxification enzymes, mediated via ARE-NRF2 pathway such as glutathione transferases, UDP-glucuronyltransferase, NAD(P)H:quinone oxidoreductase I and heme oxygenase-1 (HO-1), thereby allowing a diverse array of electrophilic and oxidative toxicants to be eliminated or inactivated before they cause damage to critical cellular macromolecules [25]. Sulforaphane has been shown to interact with KEAP1 by covalent binding to thiol groups of this inhibitory protein [26]. It was observed that sulforaphane modified multiple Keap1 domains [27], whereas the model electrophiles, but less potent pathway activators dexamethasone mesylate and biotinylated iodoacetic acid, modified Keap1 preferentially in the central linker domain [28]. Further, gene-expression profiles by an oligonucleotide microarray revealed that sulforaphane upregulated the expression of NQO1, GST and GCL in the small intestine of wildtype mice, whereas the Nrf2-null mice displayed diminished levels of these enzymes [29]. In another study, knockdown of Nrf2 with siRNA attenuated SFN-induced heme oxygenase-1 (HO-1) up-regulation [30]. *In vitro* studies have reported the time- and dose-dependent responses with sulforaphane treatment on the induction of phase II enzyme demonstrating the positive effect of 25 μM dose on the enzymatic activities of GST, NQO1, aldo-keto reductase (AKR) and glutathione reductase (GR) in several mammalian cancer cell lines: HepG2, MCF7, MDA-MB-231, LNCaP, HeLa and HT-29 [31]. Similar effects were observed in *in vivo* studies showing SFN to be effective at inducing the phase II enzyme response in rats and mice which were given SFN for four to five days at higher doses (up to 1000 mmol/kg per day), resulting in increased phase II enzyme activities in the liver, lung, mammary gland, pancreas, stomach, small intestine and colon of the animals [31]. An important aspect that need to be considered is that ROS depletion via NRF2 by any agent including sulforaphane would be eligible to block the incidence of genomic insult in order to prevent initiation of cancer, whereas the activation of the NRF2 pathway at a later stage might interfere with the efficacy of certain chemo- and radio-therapies that rely on ROS production [32].

### 2.2. Sulforaphane as Inhibitor of HDACs Challenges the Pro-Oncogenic Epigenetic Pattern in Cancer Cells

**Figure 4:**
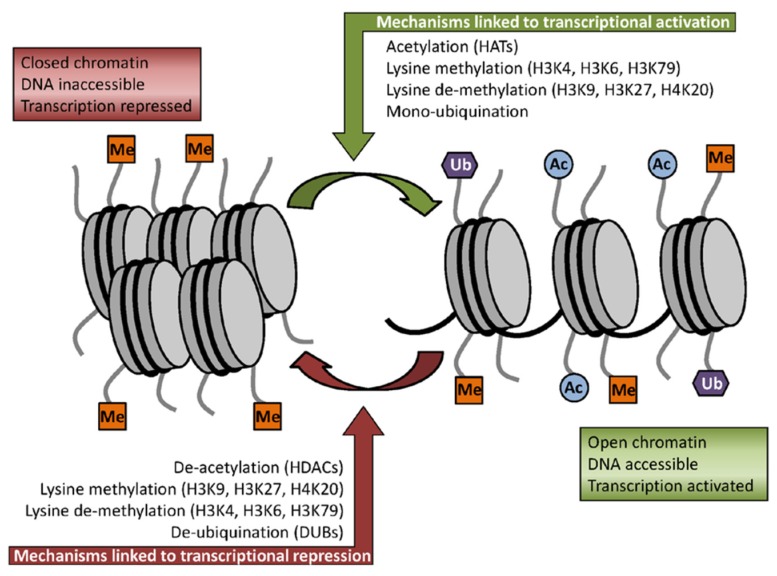
Some of the key histone modifications influencing gene expression (Me: methylation, Ub: ubiquination, Ac: acetylation). Reproduced from the original source [35] under the terms of Creative Common Attribution License.

Studies have implicated the anticancer effect of sulforaphane in its inhibitory activity against histone deacetylases (HDACs) [33] thus extending its chemopreventive activities to post-initiation stages. The mechanism of histone acetylation depends on the balance between the enzymes with histone acetyltransferase (HAT) activity and enzymes that deacetylate histones (HDACs) [34]. The reversible acetylation of nuclear histones is an important mechanism of gene regulation. Histone acetylation is associated with an open chromatin conformation, allowing for gene transcription, whereas HDACs maintain the chromatin in the closed, non-transcribed state (Figure 4) [35]. A tightly regulated balance exists in normal cells between HAT and HDAC activities, and factors influencing this balance may contribute to cancer development. These enzymes have many critical roles in regulation of gene expression, cell proliferation, cell migration, cell death and angiogenesis. HDACs are over-expressed in cancer cells making them one of the promising targets for the development of anticancer drugs [36].

It has been reported that HDAC inhibitors (HDACi) can induce growth arrest, apoptosis, reactive oxygen species facilitated cell death and mitotic cell death in cancer models [37]. Many of the studies relating sulforaphane with HDAC inhibition come from the laboratory of Dashwood and Ho [38]. In many cases, it has been established that decline in the histone acetylation state corresponds with increased grade of cancer and risk of prostate cancer recurrence [39]. Moreover, inhibitors of HDAC, including suberoylanilidehydroxamic acid (SAHA), valproic acid, depsipeptide, and sodium butyrate have been demonstrated to be effective against prostate cancer cell lines and xenograft models [40,41]. Sulforaphane treatment in prostate cellular models have shown a reduction in HDAC activity and down-regulation of HDAC proteins followed by an increase in acetylation of histone H3 at the *p21* promoter and increased acetylation of alpha-tubulin (specifically in hyperplastic and cancer cells) leading to cell death [42]. Interestingly, the enhancement in the acetylation status of alpha-tubulin demonstrates the potential of sulforaphane in the regulation of non-histone proteins also, which might have critical role in cell survival and cell death. It is known that colon cancer cells over-express HDAC3, a protein required for enhanced genomic stability. Studies have reported the ability of sulforaphane to retard HDAC3 protein expression in human colon cancer cells [43]. Another study reported that in mice treated with a single oral dose of 10 μmol SFN, there was significant inhibition of HDAC activity in the colonic mucosa and suppression of tumor development in *APC*^min^ mice (mouse model of multiple intestinal neoplasia with *APC* gene mutation) [44]. Furthermore, considering the challenge of breast cancer treatment of patients with estrogen receptor (ER)-negative tumors, studies have addressed the strategy of reactivating ERα expression and subsequent treatment with conventional anti-estrogen therapy [44,45]. The absence of ERα gene expression in ER-negative breast cancer is largely due to epigenetic silencing instead of DNA mutation or deletion of the ERα gene [45]. Treatment of ER-negative breast cancer cells with histone deacetylase inhibitors such as trichostatin A (TSA) leads to the reactivation of ER expression. HDAC inhibitors and sulforaphane epigenetically reactivates ERα expression in ERa-negative MDA-MB-231 cells. Additionally, combined treatment of green tea polyphenols and SFN along with tamoxifen therapy in hormonal refractory breast cancer significantly reduced cellular proliferation, likely due to the pronounced effect of histone modifications as well as DNA demethylation-mediated ERα activation in MDA-MB-231 cells [46].

### 2.3. Induction of Cell Cycle Arrest and Apoptosis in Cancer Cells by Sulforaphane

Sulforaphane has been shown to inhibit cell cycle progression and induce apoptosis in pre-cancerous cells and tumor cells of different origin. This anticancer agent at concentration of 75 μM was shown to cause G1/G2 cell cycle arrest and induced apoptosis by down regulating anti-apoptotic bcl-2 expression and increasing apoptosis-inducing bax expression in colon cancer Caco-2 cells [47,48]. Interestingly, another study showed that prolonged sulforaphane treatment activates survival signaling in normal NCM460 colon cells, but apoptotic signaling in HCT116 colon cancer cells [19]. It was also demonstrated that SFN (15 μmol/L) exposure (72 h) inhibited cell proliferation by up to 95% in colon cancer cells (HCT116). Moreover, SFN at doses of 5 and 10 μmol/L exhibited significant reduction of G1 phase cell distribution and induced apoptosis in cancerous HCT116 cells (but to a much lesser extent in non-cancerous NCM460 cells). Cell cycle arrest (S and G2/M) along with increased levels of transcription factors p21^WAF1^ and p27^KIP1^ and decreased levels of cell cycle regulatory proteins cyclin A, cyclin B1 and CDC2 was observed with SFN treatment of breast cancer cells MDA-MB-231 at a dose of 30 μM [49]. Sulforaphane has differential cytotoxic effects on cancer and normal cells as mentioned above in the case of colon cancer. Similarly, it was reported that SFN can more effectively inhibit the growth of MCF-7 human breast cancer cells (IC_50_ 27.9 μM) compared to MCF-12A normal human breast epithelial cells (IC_50_ 40.5 μM) for 48 h treatment [50].

Another study on acute lymphoblastic leukemia (ALL) showed normal peripheral blood mononuclear cells to be more resistant than patient lymphoblasts to sulforaphane-mediated cytotoxicity [51]. Further, treatment of ALL leukemic cells with sulforaphane resulted in dose dependent apoptosis and G2/M cell cycle arrest, which was associated with the activation of caspases (3, 8 and 9), inactivation of PARP, p53-independent upregulation of p21^CIP1/WAF1^, and inhibition of the Cdc2/Cyclin B1 complex. In the same study extending to an *in vivo* model, oral administration of sulforaphane to the ALL xenograft models resulted in a significant reduction of tumor burden. Sulforaphane could also suppress the proliferation of bladder cancer BIU87 cells with an 80 μM dose leading to the inhibition of cell proliferation, induction of apoptosis and cell cycle arrest at the G2/M phase and down regulation of pro-inflammatory NF-kB expression [52]. In a murine UMUC3 invasive bladder cell xenograft model, feeding with semi-purified diets containing four percent broccoli sprouts or two percent broccoli sprout isothiocyanate extract, or gavage of pure sulforaphane (at 295 μmol/kg; similar to dietary exposure) was reported to have resulted in tumor weight reduction by 42%, 33% and 58%, respectively [53].

**Figure 5:**
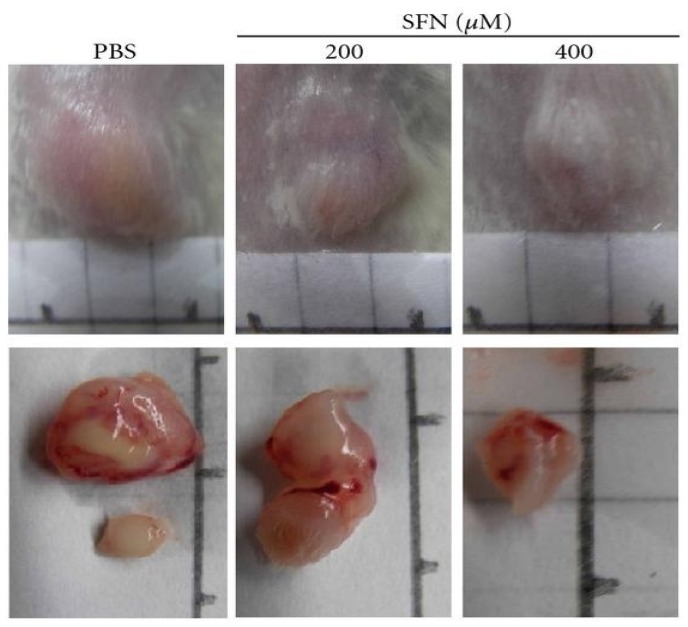
SFN inhibits *in vivo* tumor growth; Effects of SFN on tumor growth of SCID mice subcutaneously inoculated with primary human CRC cell lines. The results showed that SFN 400 μM inhibited tumor growth significantly in both cancer xenografts. Reproduced from the original source [59] under the terms of Creative Common Attribution License.

It has also been shown that sulforaphane inhibits the growth of the epithelial ovarian cancer cell (EOC) line SkOV-3 by down-regulating AKT activity [54]. Further, SFN at concentrations of 5–20 μM induced a dose-dependent suppression of growth in ovarian cancer cell lines MDAH2774 and SkOV-3 with an IC_50_ as low as ~8 μM, concomitant to increased apoptotic cell death via an increase in Bak/Bcl-2 ratio and cleavage of procaspase-9 and poly (ADP-ribose)-polymerase (PARP) [55]. In a recent study SFN was found to inhibit cell viability of both paclitaxel-sensitive (SKOV3-ip1) and -resistant (SKOV3TR-ip2) ovarian cancer cell lines time- and dose-dependently [56]. Even in high metastatic cell lines, such as salivary gland adenoid cystic carcinoma, sulforaphane induces G2-M arrest and apoptosis with a marked decline in protein levels of G(2)/M regulatory proteins including cyclin B1 and cyclin-dependent kinase 1 (CDK1) along with increased expression of Bax and down-regulation of Bcl-2 proteins [57]. An *in vivo* study using osteocarcinoma cells reported cell cycle arrest and induction of apoptosis in cells treated with SFN, as well as an effective inhibition of tumor xenograft growth [58]. In another study daily injections of SFN (400 micromol/kg/d for three weeks) in SCID mice bearing tumors derived from human primary colorectal cancer cells, resulted in a reduction of tumor weight by 70% compared to untreated mice (Figure 5) [59]. Efficacy of SFN has also been reported against human brain malignant glioma GBM 8401 cells [60] and human lung adenocarcinoma LTEP-A2 cells with growth inhibition observed in corresponding *in vivo* models [61].

### 2.4. Sulforaphane Influences Transcriptional Factors and Cellular Signaling in Cancer Cells

The mechanism-based studies have implicated a number of transcriptional factors as targets of sulforaphane leading to alteration of cellular signaling and impeding the potential of cancer cells. Sulforaphane inhibits multiple oncogenic signaling pathways often hyperactive in human cancers, including nuclear factor-κB, Akt, signal transducer and activator of transcription 3 and 5, and various survival pathway proteins. Step Miner and Ingenuity Pathway Analysis demonstrated that sulforaphane induces transcriptional changes primarily in cellular defenses and cell cycle regulation in the prostate cancer cell line LNCaP [62]. Interestingly, in time course experiments, lyophilized broccoli sprouts produced gene expression changes that were largely similar to those produced by a comparable dose of pure sulforaphane. A synthetic racemic analog of l-sulforaphane, has been shown to influence notch signaling in human prostate cancer cells PC-3 and LNCaP suggesting a therapeutic advantage [63]. The nuclear factor kappa B (NF-κB) is believed to play an important role in cancer chemoprevention due to its involvement in tumor cell growth, proliferation, angiogenesis, invasion, apoptosis, and survival. In a study on prostate cancer cells, treatment with SFN (20 and 30 μM) significantly inhibited NF-κB transcriptional activity, nuclear transloction of p65, and gene expression of NFκB-regulated VEGF, cylcin D1, and Bcl-XL in PC-3 C4 cells [64]. Studies of the molecular mechanisms of sulforaphane treatment on differentiation in the human chondrosarcoma cell line, HTB-94, demonstrated that SFN-induced enhanced expression of type II collagen, SOX-9 and phosphorylation of AKT, suggesting that SFN regulates differentiation of these cells via PI-3K/AKT [65]. It was observed that sulforaphane has inhibitory activity against myeloma cell lines and patients’ myeloma cells—both *in vitro* and *in vivo—*using a myeloma xenograft mouse model [66]. In this study, multiplex analysis of phosphorylation of diverse components of signaling cascades revealed changes in MAPK activation, increased phosphorylation of c-jun and HSP27, as well as changes in the phosphorylation of Akt, GSK3a/b and p53. The Wnt/β-catenin signaling pathway is a known regulator of cellular functions related to tumor initiation and progression, cell proliferation, differentiation, survival and adhesion. Since aberrant Wnt/β-catenin signaling has been observed in a variety of human cancers including a majority of colorectal cancers, about half of prostate cancers and a third of melanomas, dietary compounds including SFN acting as inhibitors of Wnt/β-catenin signaling are being investigated [67]. In an *in vitro* breast cancer model, sulforaphane at doses of 1–5 μmol/L decreased the aldehyde dehydrogenase-positive cell population by 65% to 80%, and reduced the size and number of primary mammospheres by eight- to 125-fold and 45% to 75%, respectively [68]. The study also reported that sulforaphane eliminated breast cancer stem cells (CSCs) *in vivo* through downregulation of the Wnt/β-catenin self-renewal pathway, thereby abrogating tumor growth after the re-implantation of primary tumor cells into secondary mice. Signal transducer and activator of transcription (STAT) factor signaling plays a critical role in cellular growth and proliferation and its activity is tightly regulated in normal cells. However, constitutively active forms of the constituent proteins of the STAT signaling pathway have been found in several human cancers including blood malignancies (leukemias, lymphomas and multiple myeloma) and solid tumors (head and neck, breast and prostate cancers) [69]. Studies have shown aberrant STAT signaling, particularly involving STAT 3 and STAT 5 proteins in the development and progression of neoplastic disease [70]. In a recent report, SFN has been shown to retard STAT5 activity, which led to the inhibition of target genes in the mouse B cell line Ba/F3 and in the human leukemic cell line K562, which express a constitutively active form of STAT5 [71].

## 3. Bioavailability and Safety Issues

A placebo-controlled, double-blind, randomized phase one study of broccoli sprout extracts, containing either glucosinolate (glucoraphanin) or ITC (sulforaphane), showed that the extracts were well tolerated and caused no significant adverse effects when administered orally at eight hour intervals for seven days at doses of 25 and 100 μmol glucosinolate or 25 μmol ITC [72]. Pharmacokinetic studies in both rats and humans demonstrated that sulforaphane can be distributed in the body and reach micromolar concentrations in the blood. Broccoli sprouts contain up to 50 times higher concentrations of the sulforaphane precursor glucoraphanin than mature broccoli, which make sprouts the better candidates for clinical trials. In human subjects given single doses of 200 μmol broccoli sprouts ITC preparation, ITC plasma concentrations peaked between 0.943 and 2.27 μmol/L 1 h after feeding, with half-life of 1.77 ± 0.13 h suggesting the possibility of clinical intervention and translational significance [73]. A recent clinical study on patients with recurrent prostate cancer showed that the administration of 200 μmol/day sulforaphane rich extracts up to 20 weeks was not associated with any adverse events [74]. Further the study also reported slight decline in PSA (<50% for 7/20 patients and >50% for 1/20 patients), and modulation of PSA doubling time with sulforaphane treatment, suggesting a possible intervention role of the phytochemical in prostate cancer prevention. Some significant clinical studies such as the POUDER trial are underway to assess the clinical plausibility of 90 mg of active sulforaphane/day as an adjuvant therapy for patients undergoing chemotherapy for pancreatic ductal adenocarcinoma [75].

## 4. Conclusions

An impressive embodiment of evidence supports the concept that dietary factors are key modulators of cancer. Epidemiological, laboratory, preclinical, and clinical studies on carcinogenesis have reflected the pharmacological significance of certain dietary agents, referred to as nutraceuticals in cancer chemprevention premises. Pleiotropic action mechanisms have been reported for diet-derived chemopreventive agents of herbal origin such as sulforaphane to retard, block or reverse carcinogenesis. In the context of their use in dietary cuisines, they are generally regarded as safe, and, in addition, provide an extended therapeutic advantage. Such alternative approaches assume significance in the light of WHO (World Health Organization) observation that recognizes traditional medicine as “an accessible, affordable and culturally acceptable form of health care trusted by large numbers of people, which stands out as a way of coping with the relentless rise of chronic non-communicable diseases in the midst of soaring health-care costs and nearly universal austerity” (WHO 2013) [76].
